# Hemicerebellitis can drive handedness shift

**DOI:** 10.1186/s40673-017-0074-6

**Published:** 2017-09-14

**Authors:** Mario Mascalchi, Matteo Lenge, Andrea Bianchi, Emanuele Bartolini, Gioele Gavazzi, Flavio Giordano, Renzo Guerrini

**Affiliations:** 1 Neuroimaging Unit, A. Meyer Children’s Hospital, University of Florence, Viale Pieraccini 23, 50139 Florence, Italy; 2 Neurology Unit and Laboratories, A. Meyer Children’s Hospital, University of Florence, Viale Pieraccini 23, 50139 Florence, Italy; 3 Pediatric Neurosurgery Unit, A. MeyerChildren’s Hospital, University of Florence, Viale Pieraccini 23, 50139 Florence, Italy

**Keywords:** Cerebellum: Childhood, fMRI, Brain mapping

## Abstract

**Background:**

Hemicerebellitisis a rare acquired condition, typical of the pediatric age. A residual switched handedness may develop after remission of acute cerebellar symptoms.

**Case presentation:**

Herein we describe a motor functional MRI studyperformed in a 35-year old girl who had switched to left-handedness after acute right hemicerebellitis in childhood. During left hand tapping, we observed activation in the right primary sensori-motor cortex, right supplementary motor area and left superior cerebellum. During right hand tapping bilateral activations of primary sensori-motorcortex and superior cerebellum including the vermis and activation of the right supplementary motor area were observed. We speculate that during right hand tapping both the ipsilateral and contralateralpre-central gyri and the ipsilateral cerebellum would be engaged in order to recover the tapping internal model of action. From this perspective the ipsilateral pre-central gyrus might serve as are transmission station of information from the healthy cerebellum to the contralateral pre-central gyrus.

**Conclusion:**

Selective damage of the right half of the cerebellum due to hemicerebellitis in childhood can drive shift of lateralized hand functions in the cerebrum.

## Background

Hemicerebellitis, namely acute inflammation of half of the cerebellum, is a rare acquired condition with obscure pathophysiology, typical of the pediatric age. It is associated with hemiatrophy of the cerebellum at follow-up [1]. At onset, most patients exhibit cerebellar symptoms, which gradually remit in 1–12 weeks. A residual switched handedness has been reported in preschool age children who suffered from right sided hemicerebellitis [1].

Hemicerebellitis may serve as a model for the possible influence of an intervening cerebellar damage in the developing brain, especially on lateralized brain functions as handedness. Functional MRI (fMRI) is a non-invasive tool to study handedness [2].

We describe herein a girl who following acute right hemicerebellitis, at 9 years of age, switched to left-handedness after having previously developed as clearly right-handed. MRI at 35 years of age showed right hemiatrophy of the cerebellum and fMRI during execution of a simple motor task were consistent with shifted handedness to the left side.

## Clinical report

### Case presentation

Family historywas negative for neurological disorders. The patient was born at term from dystocic delivery with respiratory distress (Apgar 3/8). The neonatal period was reported as normal and she acquired developmental milestones in due time. She was right-handed in childhood. At age 9 years she manifested an afebrile episode of acute unsteadiness, headache, vomiting, dysgraphia and right sided intention tremor. After unrevealing head CT scan and progressive neurological improvement over one week, she was discharged with a diagnosis of acute cerebellar syndrome. However, because of residual difficulty with use of her right hand she spontaneously began to use the left as the preferred hand for writing and other daily activities.

**Fig. 1:**
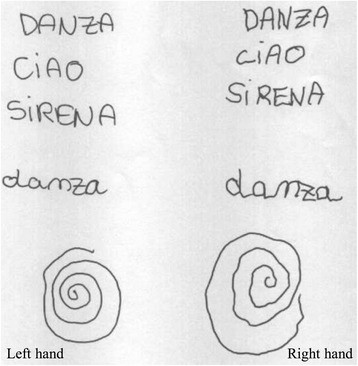
Hand-writing of three simple words and drawing the Maze spiral demonstrate preferential left handedness

At 35 years of age, neurological examination showed intention tremor of the right hand. She could write with either hand, but writing or drawing with left hand was smooth and accurate, whereas if performed with the right hand they were decomposed due to intention tremor (Fig. 1).

## Methods

**Fig. 2:**
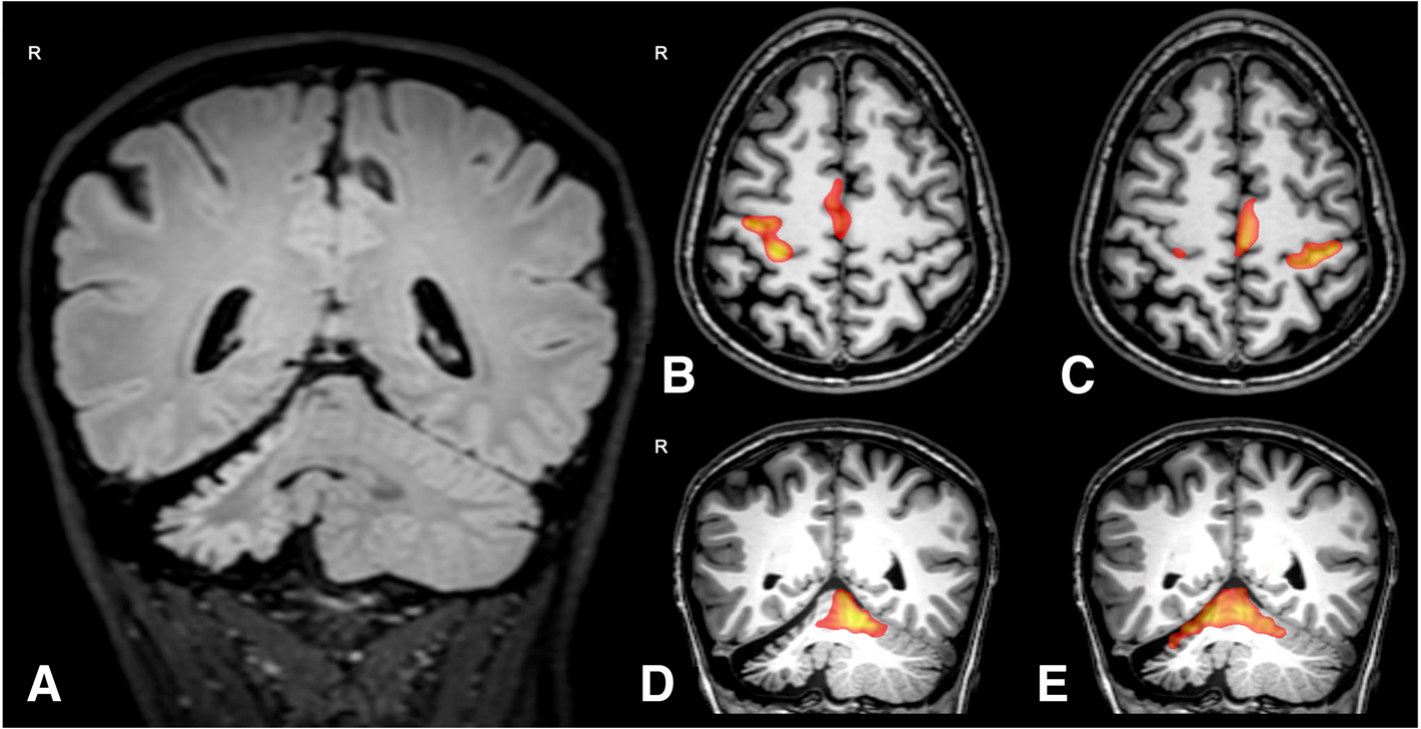
Coronal FLAIR image (**a**) shows markedly decreased volume of the right cerebellar hemisphere with hyperintensity of the right cerebellar cortex. Axial and coronal T1 weighted images with superimposed activated clusters during left (**b** and **d**) and right-hand (**c** and **e**) tapping. During left hand tapping the right precentral/postcentral gyri, right superior frontal gyrus and the left superior cerebellum are activated, whereas during right hand tapping the precentral/postcentral gyri on both sides, the left superior frontal gyrus and the bilateral superior cerebellum including the vermis are activated

Edinburgh inventory [3] confirmed left handedness (−50 score). MRI, performed at age 35 on a 3 T system (Achieva, Philips Healthcare, Netherlands equipped with a 32 channel phased-array head coil) showed a marked decrease of the volume of the right cerebellar hemisphere, with T2 high intensity signal of the right cerebellar cortex (Fig. 2). For the fMRI experiment we used a T2*- weighted echo-planar imaging (EPI) sequence (TR/TE = 3000/35 ms, FA = 90°, slice thickness = 4 mm, FOV = 230 mm × 230 mm, number of slices = 24, matrix size = 96 × 94). One hundred scans were acquired, for a total acquisition time of 5.09 min, from which the first 3 scans were discarded to avoid T1-related relaxation effects. Two fMRI experiments were performed during hand tapping with either hand. A visual cue at 1 Hz using SensaVue fMRI equipment (Invivo Corporation, Gainesville, FL, USA) was delivered through a mirror attached to the head coil.

## Results

The results of the fMRI experiments are shown in Fig. 2 and summarized in Table 1. During left hand tapping fMRI showed activation of the right primary sensori-motor cortex (SM1) and supplementary motor area (SMA) and of the left superior cerebellum. During right hand tapping there were bilateral activations of SM1 and of superior cerebellum including the vermis and activation of the right SMA.

**Table 1:** Results of fMRI experiments

Brain region	Left hand tapping											Right hand tapping										
	Right hemisphere					Left hemisphere						Left hemisphere					Right hemisphere					
	*Z*-max	X (mm)	Y (mm)	Z (mm)	N_RH_	*Z*-max	X (mm)	Y (mm)	Z (mm)	N_LH_	LI	*Z*-max	X (mm)	Y (mm)	Z (mm)	N_LH_	*Z*-max	X (mm)	Y (mm)	Z (mm)	N_RH_	LI
Precentral/postcentral	10.2	29.0	−27.8	60.3	203	–	–	–	–	0	−1	10.2	−34.0	−29.6	68.2	241	6.76	26.3	−27.4	59.2	17	0.868
Superiorfrontal	8.52	6.88	−6.13	58.6	64	–	–	–	–	0	−1	8.97	−1.74	−22.0	57.5	213	–	–	–	–	0	1
Cerebellum	–	–	–	–	0	10.8	−11.4	−52.4	−15.7	1178	−1	10.5	−13.7	−47.9	−14.4	506	11.3	14.4	−84.9	−25.9	5092	0.819

Functional cluster analysis of left and right hand fMRI tasks with correction of multiple comparison (z = 6, *P* < 0.05). Laterality index (LI) is calculated as LI = N_LH_- N_RH_ / N_LH_+ N_RH_, with N the number of voxels on left hemisphere (LH) and right hemisphere (RH) [9]. X, Y, Z are the MNI152 space coordinates of the peak

## Discussion

The more lateralized brain activation during left hand tapping in our patient is consistent with the handedness shift to the left side that was clinically observed. This finding substantially mirrors data from the fMRI study by Grabowska et al. [2] who examined right or left handers during motor tasks performed with the preferred and non preferred hand. In right handers, they observed a general predominance of left hemispheric activation with respect to activation of the contralateral hemisphere. In left handers this pattern was reversed. Interestingly, individuals who, according to a now overcome educational consuetude, had been forced to switch their left-hand preference towards the right side at an early age, termed “converted left-handers”, did not show such an asymmetry and shared features of both right-handers and left-handers.

The activated areas in the anterior cerebellum (lobule V and VI) of our patient correspond to the brain regions activated during motor tasks in healthy subjects [4]. The activation during right hand tapping of the superior vermis adjacent to the right cerebellar hemisphere may be expression of plasticity phenomena which typically involve nervous tissue bordering the damaged one [5]. Whilst the left lateralization during left hand tapping is consistent with ipsilateral cerebellar somatotopy [4], the bilateral cerebellar activation during right hand tapping could be interpreted according to the theory of internal models of actions. According to this theory, the neural representations of the external world would be stored in the cerebellum [6, 7] with the function to predict and adjust movements [8]. Therefore, during right hand tapping, bilateral pre-central gyrus and cerebellum activations in our patient might be explained in terms of a compensation process due to the acquired damage of the right cerebellar hemisphere. We speculate that during right hand tapping both the ipsilateral and contralateral pre-central gyri and the ipsilateral cerebellum would be engaged in order to recover the tapping internal model of action. In particular, from this perspective, during right hand tapping, the ipsilateral pre-central gyrus might serve as a retransmission station of information from the healthy cerebellum to the contralateral precentral gyrus.

## Conclusions

Our observation indicates that selective damage of the right half of the cerebellum due to hemicerebellitis in childhood and the corresponding compromise of lateralized cerebellar functions in the motor scheme or control can drive shift of lateralized hand functions in the cerebrum.

## Abbreviations

CT: Computed Tomography
EPI: Echo Planar Imaging
FA: Flip Angle
fMRI: Functional MRI
FOV: Field of View
MRI: Magnetic Resonance Imaging
SM1: Primary Sensory Motor cortex
SMA: Supplementary Motor Area
TE: Echo Time
TR: Repetition Time
